# Feeding, Nutrition and Rearing Systems of the Rabbit

**DOI:** 10.3390/ani13081305

**Published:** 2023-04-11

**Authors:** Marco Birolo

**Affiliations:** Department of Agronomy, Food, Natural Resources, Animal and Environment (DAFNAE), University of Padova, Viale dell’Università 16, 35020 Legnaro, Italy; marco.birolo@unipd.it

## 1. Introduction

During the last years, several issues have contributed to a progressive decline in rabbit meat consumption in the European Union, including consumers’ concerns for animal welfare, the unsuitable presentation of the end product, an increased popularity of rabbits as pet animals, high production costs (aggravated by the ongoing geopolitical crises), and criticism about the environmental sustainability of rabbit farms [1]. Therefore, the rabbit sector is looking for sustainable solutions to limit the use of antimicrobials, improve the health and welfare of animals, and reduce the environmental impact of farms. Progress in genetics, breeding management, and feeding techniques have enhanced health conditions and feed efficiency in rabbit production; however, further improvements are required to guarantee the economic sustainability of rabbit farms [2,3].

This Special Issue of *Animals* collects research papers and literature reviews on genetic, feeding, and nutritional strategies for improving health, feed efficiency, and meat quality in growing rabbits. Innovative techniques for the determination of nutrients use efficiency have also been investigated.

## 2. Recent Advances in Rabbit Production in This Special Issue

The microbiota hosted in the gastrointestinal tract of the rabbit is involved in several crucial roles, including the regulation of digestive processes, stimulation of the immune response, and contrasts to biotic and abiotic stresses. According to the available information, the use of probiotics in growing rabbits can positively affect intestinal morphology, modulate the composition of gastrointestinal microbiota, and reduce mortality and morbidity rates [4]. Some benefits on growth performance and digestive and feed efficiency have also been reported. Studies on this topic are particularly relevant to provide socially and environmentally friendly alternatives to the use of antimicrobials and to improve profitability in rabbit production. However, further research efforts are required to develop suitable technologies for the dietary supplementation of probiotics that need to reach the target site of action to exert their beneficial effects [4].

In a recent study, dietary supplementation with lysozyme has been effective in enhancing rabbits’ growth, caecal fermentation, blood lipid profile, and antioxidant status [5]. Furthermore, alternative ingredients such as *Moringa oleifera* leaves have been tested in the diets of growing rabbits, with positive effects on growth, feed conversion ratio, antioxidant status, and meat fatty acids profile [6]. Testing alternative feedstocks is particularly important to sustain the expansion of the rabbit industry in developing countries where conventional raw materials are less available or not available at all. In addition, *M. oleifera* leaves are rich in essential nutrients and bioactive compounds that can improve the nutritional profile of rabbit meat [6].

The use of high-energy diets in the post-weaning period, provided that minimal insoluble fibre contents are guaranteed, has proven to be a viable strategy to improve feed conversion ratio and reduce feeding costs in rabbit production [7]. The use of fatty ingredients such as soybean oil is a common strategy to increase the digestible energy content of diets. Nonetheless, recent findings have shown that lard and palm kernel oil may be valuable alternative sources of fat as they have been associated with reduced mortality in growing rabbits [8]. An increase in dietary fat content can also be useful to improve nitrogen use efficiency and limit nitrogen excretion in growing rabbits [8]. Moreover, when diets are supplemented with essential amino acids according to the current recommendations, dietary crude protein levels can be reduced without affecting the growth and feed efficiency of high-producing fattening rabbits [7]. On the other hand, a low-quality protein (i.e., protein with an unbalanced amino acid profile) in the diet negatively affects productive and reproductive traits, and increases nitrogen excretion. Thus, the development of tools for an early detection of poor nutrients use efficiency is fundamental to improve the environmental sustainability of rabbit farms. Urea nitrogen seems to be an appropriate indicator of amino acid imbalance in monogastric animals, including rabbits [9]. Furthermore, bioelectrical impedance has been proposed as a non-invasive method to estimate in vivo carcass composition and to determine the nutrient retention and overall energy and nitrogen retention efficiencies of fattening rabbits [10]; however, more studies on these topics are required.

Genetic selection can be applied not only to optimize the growth performance and feed efficiency of growing rabbits but also to create lines with different meat attributes, such as fat lines to provide greater amounts of healthy fatty acids, and lean lines to provide lean meat for everyday consumption. A divergent selection for total body-fat content measured by computed tomography has been effective in creating two rabbit populations (fat and lean) for different production purposes [11]. This study will support the adoption of genetic selection programs aimed at diversifying rabbit production in terms of carcass and meat composition according to consumer demands.

## 3. Conclusions

The future of the rabbit sector depends on its capability to address the ongoing global challenges and offer safe, highly nutritional, and environmentally and ethically sustainable products at affordable prices. Each of the research topics published in this Special Issue has significantly contributed to increasing the state of knowledge on rabbit genetics, feeding, and nutrition, opening innovative routes that should be explored to enhance sustainability in rabbit production.
